# A Case of Bilateral Transient Pregnancy-Related Osteoporosis of the Hip Diagnosed Late During the Lactation Period: A Rare Clinical Presentation and a Mini Review of the Literature

**DOI:** 10.7759/cureus.63509

**Published:** 2024-06-30

**Authors:** Efthymia Thanasa, Anna Thanasa, Ioannis-Rafail Antoniou, Emmanouil M Xydias, Alexandros Leroutsos, Gerasimos Kontogeorgis, Ioannis Paraoulakis, Apostolos C Ziogas, Ioannis Thanasas

**Affiliations:** 1 Department of Health Sciences, Medical School, Aristotle University of Thessaloniki, Thessaloniki, GRC; 2 Department of Obstetrics and Gynecology, General Hospital of Trikala, Trikala, GRC; 3 Department of Obstetrics and Gynecology, EmbryoClinic IVF, Thessaloniki, GRC; 4 Department of Obstetrics and Gynecology, University of Thessaly, Larissa, GRC

**Keywords:** transient osteoporosis, pregnancy, lactation, magnetic resonance imaging, bisphosphonates, prognosis, case report

## Abstract

Transient pregnancy-related osteoporosis of the hip is a rare, idiopathic, benign, and usually self-limiting condition caused by edema of the bone marrow, which can be visualized on magnetic resonance imaging. Bilateral localization of the disease is even less common. Our case concerns a 31-year-old primigravida who, during the 35th week of pregnancy, was hospitalized at the Obstetrics and Gynecology Clinic of the General Hospital of Trikala with lumbar and hip pain. The pain gradually increased in intensity and was accompanied by severe movement limitation. No history of falls or injury was reported. Her personal history was unremarkable, and the course of the pregnancy was uneventful. A clinical examination by a team of orthopedic surgeons established a diagnosis of acute hip and back pain. Rest and administration of paracetamol did not improve her clinical condition. During the postpartum and lactation period, the lack of symptom relief led to the decision to further evaluate the patient. The diagnosis of pregnancy-related transient osteoporosis of both hips was established by magnetic resonance imaging. Immediate treatment with bisphosphonate medication after the discontinuation of breastfeeding led to a definitive remission of the symptoms three months later. In this study, after the case description, a brief literature review of this rare clinical entity is presented. Proper knowledge of this condition helps to provide the best possible short- and long-term prognostic outcomes for the mother, fetus, and newborn.

## Introduction

Osteoporosis is a chronic condition that is a major public health concern. The increasing incidence of osteoporosis observed in recent years, due to an aging population, affects millions of people around the world and results in a significant economic burden [1]. Osteoporosis, resulting from a combination of genetic, dietary, hormonal, age, and lifestyle-related factors, is characterized by low bone density and dysfunction of bony tissue. This leads to reduced bone strength, increased bone fragility, and an elevated risk of fractures in the hip, spine, and other skeletal sites [1,2]. Osteoporosis typically affects individuals older than 50 years of age. It is estimated that one in three women and one in five men older than 50 years will experience fractures associated with osteoporosis [3]. It is also estimated that pregnant women or women during lactation may be affected by the disease, resulting in vertebral fractures, height loss, and further spinal kyphosis [4].

Normally progressing pregnancy and the lactation period are characterized by a slight loss of bone density [5]. Pregnancy causes physiological changes in calcium levels and hormones that regulate calcium homeostasis. In pregnant women, there is a decrease in serum calcium, which is caused by decreased albumin levels. In contrast, levels of ionized calcium, phosphorus, parathyroid hormone, and calcitonin do not appear to be affected by pregnancy and remain unchanged. Additionally, the significant hormonal changes, particularly in estrogen and prolactin that normally occur in the maternal organism during pregnancy and breastfeeding may lead to a loss of bone density. Depending on its extent, this loss may cause osteoporosis or skeletal fractures. Furthermore, during pregnancy, intestinal absorption of calcium doubles to compensate for the needs of the growing fetal skeleton [6,7].

The description of bilateral transient pregnancy-associated hip osteoporosis as a rare obstetric complication with unknown short- and long-term effects is the main focus of the authors. Concurrently, this paper emphasizes the value of early diagnosis of osteoporosis during pregnancy and the significant diagnostic difficulties that may delay early and accurate diagnosis of the condition.

## Case presentation

The case concerns a 31-year-old primigravida who presented at the outpatient clinic of the Obstetrics and Gynecology Clinic of the General Hospital of Trikala during the 35th week of pregnancy, complaining of pain in her left hip area. The pain, moderate in intensity, began approximately 10 days prior without any reported fall or injury. Her personal history was unremarkable, and the pregnancy had been progressing without complications. Since the first trimester, she has been taking iron and calcium supplements. Her body mass index was 32. The pregnancy was spontaneous, and she did not smoke nor did she have a family history of osteoporosis. Initially, she was advised to rest and avoid physical activity and was scheduled for a follow-up examination. However, three days later, the pain intensified significantly, spreading to her right hip area, lumbar region, and bilaterally along her lower limbs. The pain was now severe and accompanied by substantial restriction of movement. The patient reported needing substantial assistance to get out of bed and was almost unable to walk independently.

The pregnant woman was admitted to the clinic. Obstetric clinical examination and laboratory tests revealed no abnormal findings (Table 1).

**Table 1:** Laboratory control of the patient during her hospitalization in the clinic before the cesarean section and one month after the cesarean section. Ht: hematocrit; Hb: hemoglobin; PLT: platelets; WBC: white blood cells; NEUT: neutral; APTT: activated partial thromboplastin time; INR: international normalized ratio; CRP: C-reactive protein; Glu: glucose; Cr: creatinine; Na+: sodium; K+: potassium; Alb: albumin; ALP: alkaline phosphatase; Ca: calcium; Mg: magnesium; P: phosphorus; Vit D: vitamin D.

Laboratory tests	Day of admission to the clinic	48 hours of hospitalization	30 days after cesarean section	Normal laboratory values
Ht	37.7%	36.9%	38.7%	37.7 – 49.7%
Hb	12.4 gr/dl	12.1 gr/dl	12.9 gr/dl	11.8 – 17.8 gr/dl
PLT	210x10^3^/ml	215x10^3^/ml	220x10^3^/ml	150 – 350 x10^3^/ml
WBC	9.3x10^3^/ml	9.1x10^3^/ml	8.7x10^3^/ml	4 – 10.8 x10^3^/ml
NEUT	73%	74%	69%	40 – 75%
APTT	28.3 sec	28.1 sec	29.4 sec	24.0 – 35.0 sec
INR	0.99	0.97	0.96	0.8 – 1.2
CRP	0.1 mg/dl	0.15 mg/dL	0.2 mg/dL	0.5 mg/dl
Glu	98 mg/dl		87 mg/dl	75 – 115 mg/dl
Cr	0.51 mg/dl		0.62 mg/dl	0.40 – 1.10 mg/dl
Na^+^	136 mmol/L		141 mmol/L	136 – 145 mmol/L
K^+^	4.3 mmol/L		3.9 mmol/L	3.5 – 5.1mmol/L
Alb	3.9 g/dl		4.7 g/dl	3.4 – 4.8 g/dl
ALP	119 U/L		94 U/L	25 – 125 U/L
Ca	8.4 mg/dl		8.8 mg/dl	8.1 – 10.4 mg/dl
Mg	1.7 mg/dl		1.7 mg/dl	1.6 – 2.3 mg/dl
P	4.1 mg/dl		4.2 mg/dl	2.6 – 4.5 mg/dl
Vit D	47 ng/dl		49 ng/dl	7 – 53 ng/dl

A team of orthopedic surgeons clinically examined her and diagnosed acute sciatica. Rest, systemic pain management, and termination of pregnancy via cesarean delivery were recommended. Administration of paracetamol did not alleviate the pregnant woman's symptoms. She underwent cesarean section delivery at 38 weeks of gestation (37 weeks and three days) after spontaneous onset of labor, delivering a live, mature male infant weighing 3100 grams. On the fourth day post surgery, the patient was discharged from the clinic. Her musculoskeletal symptoms were slightly improved compared to during pregnancy.

**Figure 1:**
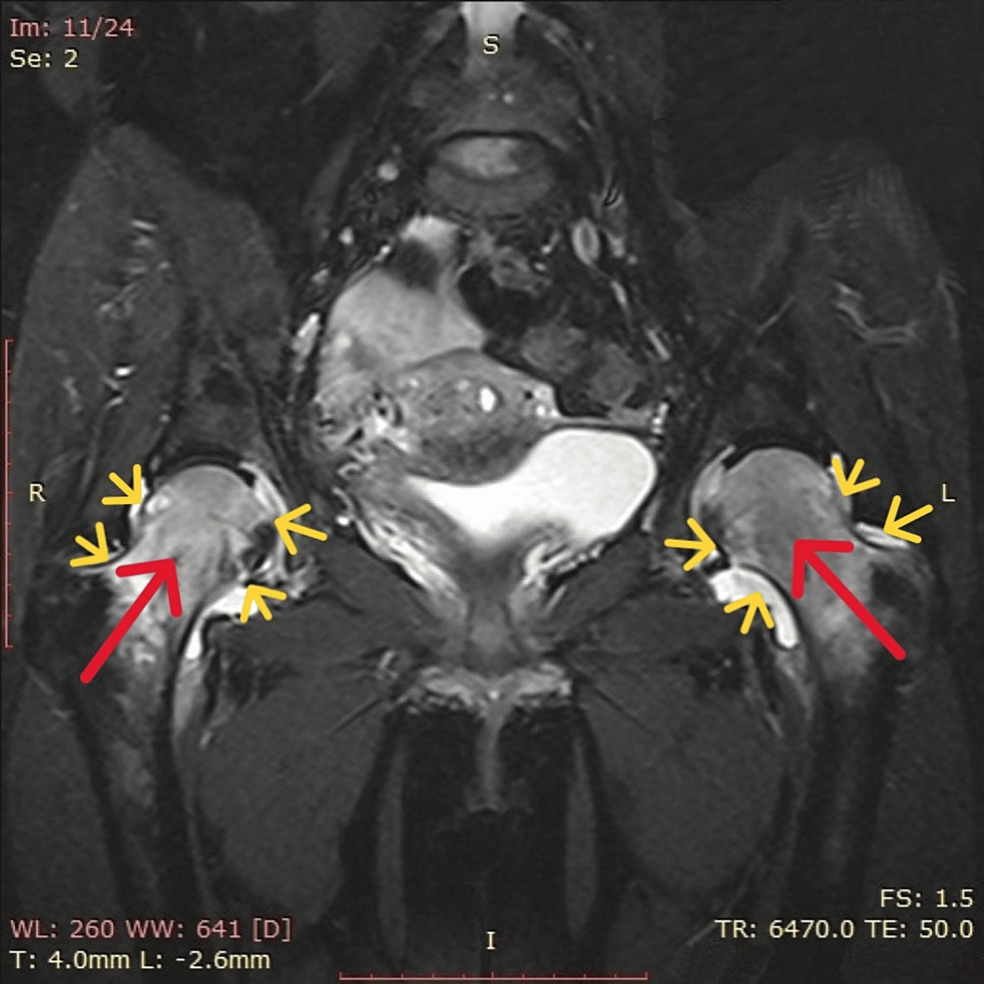
Magnetic resonance imaging of bilateral transient pregnancy-related osteoporosis of the hip. MRI showing intraosseous edema of the bone marrow in both femoral heads, extending to the necks of both femurs (red arrows), along with a moderate fluid collection in the hip joints (yellow arrows).

During pregnancy and lactation, the lower back and leg pain persisted. Despite notable improvement in clinical symptoms and moderate pain relief with analgesic and anti-inflammatory agents, the incomplete resolution of symptoms prompted further evaluation. One month after the cesarean section, repeat hematological and biochemical tests showed normal values (Table 1). Similarly, imaging examinations, including X-rays of the lumbar spine, pelvis, and hip region, revealed no abnormalities. The diagnosis of pregnancy-related transient osteoporosis affecting both hips was confirmed through magnetic resonance imaging. The study indicated diffuse intraosseous edema in the bone marrow of both femoral heads, extending to the necks of both femurs up to the intertrochanteric line bilaterally. Additionally, moderate fluid accumulation was observed in both hip joints (Figure 1).

**Figure 2:**
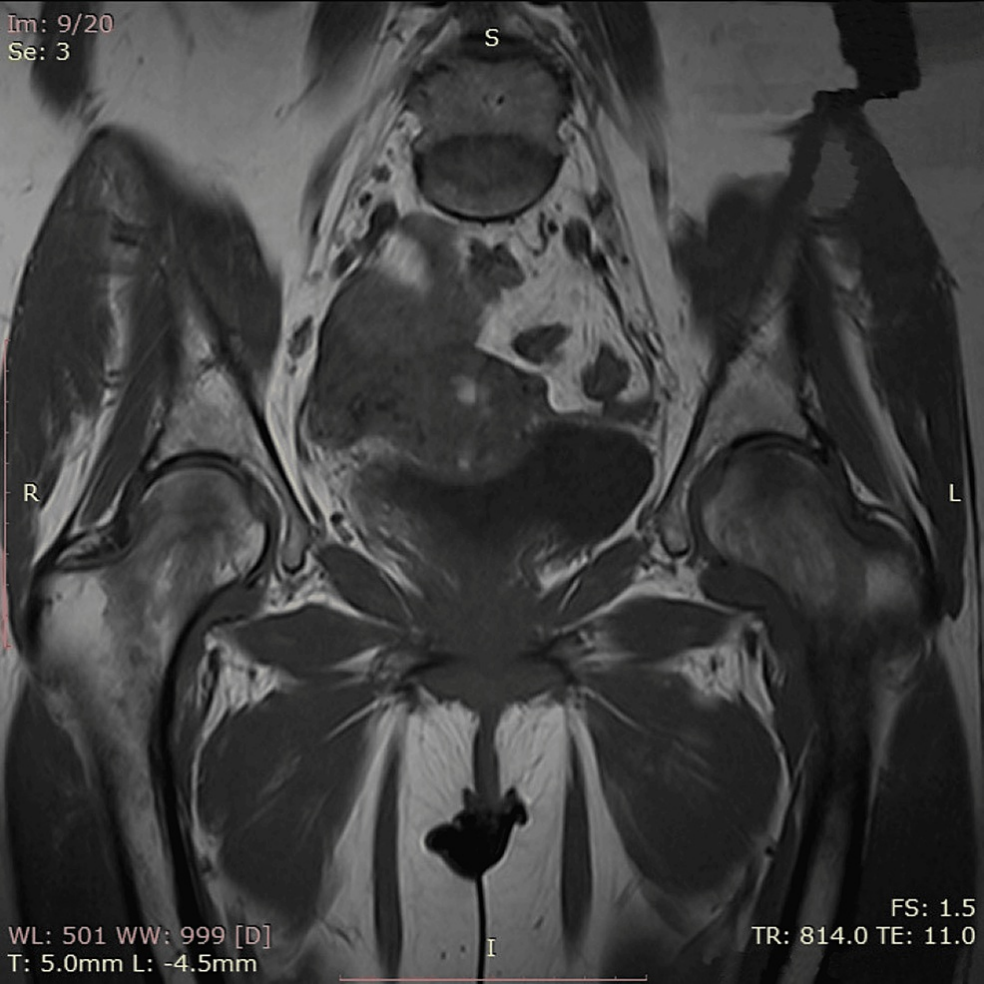
Magnetic resonance imaging of bilateral transient pregnancy-related osteoporosis of the hip. After the administration of intravenous contrast material, no abnormal enhancement of the bone marrow in either femur was detected. This absence of abnormal enhancement rules out the presence of tumors (primary or metastatic), infections (osteomyelitis), or other inflammatory conditions.

Following administration of intravenous contrast material, no abnormal enhancement of the bone marrow in either femur was detected (Figure 2).

Immediate initiation of oral bisphosphonate therapy after pharmacological cessation of lactation led to the complete resolution of the patient's symptoms three months later.

## Discussion

Transient pregnancy- and lactation-related osteoporosis is a severe type of premenopausal osteoporosis that occurs in the second and especially in the third trimester of pregnancy, or immediately after childbirth during the postpartum period. It usually occurs during the first pregnancy and does not recur in subsequent pregnancies [8]. In the majority of cases (76%), the hip joints are affected. Less commonly, the condition may affect the knees, feet, and ankles [9]. Although no case of bilateral localization of the condition in the hips has been described outside pregnancy, it can be observed in pregnant women (as in our case) [10]. Transient osteoporosis of the hip is a rare and benign skeletal disorder whose etiology and overall incidence remain relatively unknown [11]. It was first described as a distinct disease entity by Nordin and Roper in 1955 [12]. The first three cases of transient osteoporosis of the hip in pregnancy were reported in 1959 by Curtiss and Kincaid [13]. From 1955 to 2006, about 100 cases of osteoporosis associated with pregnancy and lactation have been described in the English literature [14]. The incidence of pregnancy- and lactation-related osteoporosis is estimated to be one case per 250,000 pregnant women, without taking into account the number of undiagnosed cases, which is estimated to be quite high [15,8].

The exact etiopathogenetic mechanism of pregnancy-related osteoporosis remains unknown. Although several cases have been reported in the international literature, the etiology of transient pregnancy-related osteoporosis of the hip is poorly understood. This is mainly due to the lack of systematic analyses to date, which could clarify the etiopathogenesis of the disease [16]. Pregnancy-related transient hip osteoporosis is thought to be a multifactorial disease, which may be related to immobility, dental problems, and lack of exercise in childhood [17]. Recently, in 2023, Toussia-Cohen et al., analyzing the results of their study, which included a series of patients with unilateral or bilateral transient osteoporosis of the hip during pregnancy or postpartum, showed that women diagnosed with transient osteoporosis of the hip were advanced in age, had a low body mass index, and no family history of osteoporosis. They also showed that these women were smokers and that their pregnancy was the result of in vitro fertilization [18]. Typically, our patient had none of the risk factors for osteoporosis in pregnancy: our patient was not of advanced age, had never been a smoker, had a high body mass index, had no family history of osteoporosis, and had conceived spontaneously.

The clinical diagnosis of transient osteoporosis in pregnancy is challenging. Difficulties in diagnosis are largely due to the fact that there are no specific risk factors associated with the development of the condition in pregnant women [19]. Furthermore, the relatively young age of these patients and the possible overlap of mild disease symptomatology with the physiological changes and pain frequently reported by pregnant women during normally progressing pregnancy present additional diagnostic difficulties [20]. The characteristic clinical feature of transient osteoporosis in pregnancy is pain in the lower thoracic and/or lumbar region (spinal form of the disease) or hip pain in cases of transient pregnancy-related hip osteoporosis (as in our case). In many cases, hip movement is limited by a rapid onset of severe and persistent hip pain. This pain may extend to the groin and thigh, causing significant limitation of mobility and fractures [9]. In isolated cases of transient hip osteoporosis in pregnancy, femoral neck fracture may occur, which is a rare complication [21]. Transient osteoporosis of the hip in pregnant women can be difficult to differentiate from other causes of hip pain, such as vascular necrosis, septic arthritis, and malignancy, and is often disregarded as musculoskeletal pain without further diagnostic investigation [22,23]. Misdiagnosis as sciatica may be common [24]. It is not surprising that in our patient, the hip pain, given the significant weight gain during pregnancy, was initially attributed to musculoskeletal causes, considered as lumbar sciatica, and no imaging investigation was deemed necessary.

Additionally, assessing bone density in pregnant women poses fundamental challenges. The exposure of the fetus to radiation emitted by bone density measuring equipment, and the fact that both body weight and soft tissue constitution affect the measurement, make it difficult to evaluate bone density in pregnant women despite the application of modern techniques [25]. Plain radiographs can detect affected joints and may highlight severe osteopenia in advanced stages of hip osteoporosis [22]. In contrast, magnetic resonance imaging is now considered the modality of choice for diagnosing transient pregnancy-related osteoporosis of the hip. Low or intermediate signal intensity in T1-weighted sequences and high signal intensity in T2-weighted sequences support the diagnosis of bone edema due to increased water content in the bone marrow [26]. In the case of fracture, a thin line of low signal is visualized in the bone region, and the surrounding area shows a low signal in T1-weighted sequences and a high signal in T2-weighted sequences with an area of edema [22]. Bone edema on magnetic resonance imaging is a characteristic finding and is detected with high sensitivity even 48 hours after the onset of symptoms [27,28]. However, it is noted that magnetic resonance imaging findings can often be inconclusive and create serious problems in the differential diagnosis between transient pregnancy-related hip osteoporosis and early vascular necrosis [29].

Effective treatment of pregnancy-related osteoporosis is controversial. The self-limiting nature of most cases of the disease often renders the decision on the timing of initiation and the type of medication complex. Some consider the implementation of treatment unnecessary since a progressive increase in bone mass occurs gradually in most pregnant women with transient osteoporosis of the hip [17,30]. Furthermore, the lack of robust comparative research data to date prevents drawing safe conclusions about the optimal therapeutic intervention for patients with transient pregnancy- and lactation-related osteoporosis [31]. Avoiding or discontinuing breastfeeding, avoiding weight lifting, moving with the use of a wheelchair or assistive devices, applying physiotherapy to prevent contractions of the involved hip muscles, and administering mild analgesic drugs such as paracetamol and mild non-steroidal anti-inflammatory drugs are conservative first-line treatment options aimed at relieving pain and preventing microfractures [32]. The subsequent administration of drugs, such as calcitonin, teriparatide, and denosumab, forms the basis of treatment for pregnant women with osteoporosis, as these are among the safest treatment options. They are not incorporated into the bone and therefore have a lower theoretical long-term risk [30]. Oral or intravenous administration of bisphosphonates should be selected with caution in pregnant women. Studies have shown that bisphosphonates, although providing significant improvement in symptoms and bone density in patients with pregnancy- and lactation-related osteoporosis, can affect fetal skeletal development, causing preterm delivery, fetal growth restriction, transient neonatal hypocalcemia, and spontaneous abortion [33]. Additionally, the mode of delivery should be chosen with particular care. Although there is no documented contraindication in the literature for normal delivery, the restriction of hip movements caused by pain makes it necessary in most cases to perform an elective cesarean section to protect these women from delivery-related injuries [34]. In our patient, oral treatment with bisphosphonate drugs was chosen after the medical cessation of breastfeeding, since the diagnosis was made late after the birth of the fetus. Additionally, the bilateral localization of the disease, the intensity of symptoms, and our patient's desire for future pregnancy were additional factors for initiating medication for transient pregnancy-related hip osteoporosis.

The prognosis of pregnancy-related transient osteoporosis is usually favorable. Pregnancy- and lactation-associated osteoporosis is a benign, self-limited condition characterized by the spontaneous regain of lost bone mass without treatment within the first two months after delivery [9]. Also, magnetic resonance imaging findings in the majority of cases return to normal within three to six months after delivery [35]. The disorder is not associated with recurrence in future pregnancies [8]. Cases in which symptoms persist for several years are rare. Long-term effects are not typical of the disease, except in those cases complicated by severe bone fractures [36,37].

## Conclusions

Bilateral transient pregnancy-related osteoporosis of the hip is a rare clinical entity that should be considered in the differential diagnosis of patients with lower lumbar and/or hip pain during pregnancy or postpartum. Early diagnosis and treatment are particularly important in preventing osteoporotic fractures and improving the quality of life of these patients. Disease-related fractures can be a major cause of long-term disability. In the absence of definitive insight into the pathophysiological mechanisms responsible for the disease, future prospective clinical trials are essential to increase our knowledge of current evidence-based approaches for early and effective diagnosis and treatment of pregnancy- and lactation-related transient osteoporosis.
